# Supplementary material for: Molecular identification and antimicrobial potential of endophytic fungi against some grapevine pathogens
Source: PLoS One. 2024 Oct 24;19(10):e0309041. doi: 10.1371/journal.pone.0309041 (PMC11501011; doi:10.1371/journal.pone.0309041)
Supplement: Attachment — Submitted filename: Response to reviewers.pdf [file pone.0309041.s003.pdf]

The responses to the comments are given below.

Reviewer #1:.

1)Line 14- 15. The endophyte P. maximus is not included in the 12 isolates, then why it was shown as the most effective inhibitor of grapevine pathogen in the manuscript. This strain was not shown in either the abstract or in the tables.

Author response:-

Table (1) presents the identification of twelve endophytic fungi based on the analysis of ITS (Internal Transcribed Spacer) and SSU (Small Subunit) sequences. Other endophytes, including *P. maximus*, *A. alternata*, *Penicillium radiatolobatum*, and *C. buchloes*, were identified solely through ITS sequencing. These endophytic fungi were employed as biological agents against plant pathogenic fungi.

To address the reviewer's concern, there are two options: either to add *P. maximosus* to the abstract and relevant tables or to remove it from the biocontrol analysis. I would appreciate the reviewer's guidance on which approach would be most suitable

2)Some pathogenic fungi causing plant diseases were also isolated such as *B.dothidea*, and *F.oxysporum.* How these can be used as the biocontrol agents?

Author response:-

Although *B. dothidea* and *F. oxysporum* are primarily known as plant pathogens, their potential as biocontrol agents underscores the complexity of fungal interactions within plant ecosystems. Non-pathogenic strains of *Fusarium oxysporum* and *B. dothidea* have demonstrated biocontrol potential against various plant diseases in previous studies. For instance, Larkin and Fravel (2002) showed that a non-pathogenic strain of *F. oxysporum* effectively controlled Verticillium wilt in potato plants. Additionally, Meenu Katoch et al. (2014) reported that *B. dothidea*, when isolated as an endophytic fungus, can serve as a potential biocontrol agent.

3)Line 45-48. Please provide more citations explaining the endophytic antagonistic effects against B.ceneria and I.destructans, two of the most destructive pathogens of grapevine.

Author response:-

Provided more citations explaining the endophytic antagonistic effects against B.ceneria and I.destructans, two of the most destructive pathogens of grapevine.

4)Lines# 96-107. Provide details and quantities of PCR reaction mixture for both ITS and SSU.

Author response:-

quantities of PCR reaction mixture for both ITS and SSU included:

12.5 µl of Taq PCR master mix (Add Bio, Korea), 1 µl of both forward and revers primer of 10 pmol, 2 µl genomic DNA, and 8.5 µl RNase free water in 25µl of final volume reaction.

5)Several grammatical errors were found in lines # 116- 159.

Author response:-

Corrected

6)Table 1 needs formatting. It's not the correct style.

Author response:-

Corrected

7)Grammatical errors in lines# 170- 185.

corrected

8)Fig. 1. Species names must be in italics. Provide reference to the NJ method.

Author response:-

Provided

9)Line#207. Where is P.maximus in Table 1?

Author response:-As responded in a comment (1).

"This study is part of a PhD project. *P. maximus, A. alternata,* and *C. buchloes* were identified only through ITS sequencing; therefore, they are not included in the table and are not mentioned in this manuscript. This manuscript (in the molecular study) includes only the fungi identified based on both ITS and SSU primers.

The table below shows the fungi identified based on ITS primers only and not included in this study (in the Molecular identification section).

Scientific name	Per identity	Accession number
Paecilomyces maximus	100%	PP410469
Alternaria alternata	99.81%	PP410473
Curvularia buchloes	100.00%	PP410464
Penicilium radiatolobatum	99.825	PP410466

10)Table 3. Not the correct style. Needs formatting.

Author response:-

Corrected

11)Fig 2 needs major revision. No horizontal and vertical titles, no error bars.

Author response:-

Corrected

12)The results and findings have not been discussed properly in the light of previous literature. Only four references were mentioned to support the results of the present study.

corrected

Reviewer #2:

Abstract

1. Line 2-5- The geographical location of the study location must be known eg. Nigeria etc

Author response:-

Added >>>> It is "Duhok Province in the Kurdistan Region of Iraq"

2. A brief introduction to the subject must be given in 2 lines

Author response:-

Added

3. Line 9- " These fungi were "Intro

Author response:-

corrected

4. Line 51- Our study sought to....."

Author response:-

corrected

Methods

5. More than 1 medium should have been used to plate the samples since it creates more diversity in species of fungi. A better picture is given…

Author response:-

Two media were used in our study: Potato Dextrose Agar (PDA) and Malt Extract Agar (MEA). As suggested by the reviewer, we included both media in our methodology. We did not initially mention MEA in the method section because the same fungi were isolated from both PDA and MEA.

6. Results from this present study must be compared with similarly one works from across the globe to ascertain differences and possible sound scientific reasons given.

Author response:-

corrected

7. PLOS authors have the option to publish the peer review history of their article (what does this mean?). If published, this will include your full peer review and any attached files.

Author response:- Yes
